# Treatment of Large Periapical Cyst Like Lesion: A Noninvasive Approach: A Report of Two Cases

**DOI:** 10.5005/jp-journals-10005-1299

**Published:** 2015-08-11

**Authors:** Nikhil Sood, Neha Maheshwari, Rajat Gothi, Niti Sood

**Affiliations:** Reader, Department of Conservative Dentistry and Endodontics Vananchal Dental College, Garhwa, Jharkhand, India; Postgraduate Student, Department of Pedodontics and Preventive Dentistry, SGT Dental College, Gurgaon, Haryana, India; Postgraduate Student, Department of Periodontics, Subharti Dental College, Meerut Uttar Pradesh, India; Senior Lecturer, Department of Prosthodontics, Vananchal Dental College Garhwa, Jharkhand, India

**Keywords:** Calcium hydroxide, Healing, Metapex, Periapical lesion.

## Abstract

Periapical lesions develop as sequelae to pulp disease. Periapical radiolucent areas are generally diagnosed either during routine dental radiographic examination or following acute toothache. Various methods can be used in the nonsurgical management of periapical lesions: the conservative root canal treatment, decompression technique, active nonsurgical decompression technique, aspiration-irrigation technique, method using calcium hydroxide, lesion sterilization and repair therapy and the apexum procedure. Monitoring the healing of periapical lesions is essential through periodic follow-up examinations. The ultimate goal of endodontic therapy should be to return the involved teeth to a state of health and function without surgical intervention. All inflammatory periapical lesions should be initially treated with conservative nonsurgical procedures. Surgical intervention is recommended only after nonsurgical techniques have failed. Besides, surgery has many drawbacks, which limit its use in the management of periapical lesions.

**How to cite this article:** Sood N, Maheshwari N, Gothi R, Sood N. Treatment of Large Periapical Cyst Like Lesion: A Noninvasive Approach: A Report of Two Cases. Int J Clin Pediatr Dent 2015;8(2):133-137.

## INTRODUCTION

Periapical lesions are sequelae to endodontic infection caused due to dental caries or trauma and manifest itself as the host defense response to microbial challenge emanating from the root canal system. It is viewed as a dynamic encounter between microbial factors and host defenses at the interface between infected radicular pulp and periodontal ligament that results in local inflammation, resorption of hard tissues, destruction of other periapical tissues.^1,2^

Large periapical lesions are often associated with anterior maxillary teeth, probably due to traumatic injuries. These lesions could be classified as granulomas, pocket cysts (also called as bay cysts) and true cysts. Granulomas usually composed of solid soft tissue, while cysts have semi solid or liquefied central area usually surrounded by epithelium.^3^ Pocket cysts have an epithelium lining that is connected with the root canal and true cysts are completely lined with epithelium and not connected with the root canal.^3^

It is generally accepted that periapical lesions cannot be differentially diagnosed as either radicular cysts or apical granulomas based on radiographic evidence alone. The incidence of cysts within periapical lesions varies between 6 and 55%. The occurrence of periapical granulomas ranges between 9.3 and 87.1%, and of abscesses between 28.7 and 70.07%.^4^ Natkin et al stated that with a radiographic lesion size of 200 mm^2^ or larger, the incidence of cysts was almost 100%. If the lesion is separate from the apex and with an intact epithelial lining (apical true cyst) it may have developed into a self-perpetuating entity that may not heal when treated nonsurgically.^5^

On other occasions, a large periradicular lesion may have a direct communication with the root canal system (apical pocket cyst) and respond favorably to nonsurgical treatment. Clinical studies have confirmed that simple nonsurgical treatment with proper infection control can promote healing of large lesions. When this treatment is not successful in resolving the periradicular pathosis, additional treatment options should be considered, such as marsupialization or tube decompression.^5^

However, a preliminary clinical diagnosis of a peri-apical cyst can be made based on the following: (a) The periapical lesion is involved with one or more non-vital teeth, (b) the lesion is greater than 200 mm^2^ in size, (c) the lesion is seen radiographically as a circumscribed, well-defined radiolucent area bound by a thin radiopaque line, and (d) it produces a straw-colored fluid upon aspiration or as drainage through an accessed root canal system.^6^

The following case reports describe the management of a particularly large maxillary periapical lesion (involving anterior teeth) by draining the abscess followed by nonsurgical endodontic treatment using interim long-term calcium hydroxide [Ca(OH)_2_].

## CASE REPORTS

### Case 1

**Fig. 1:**
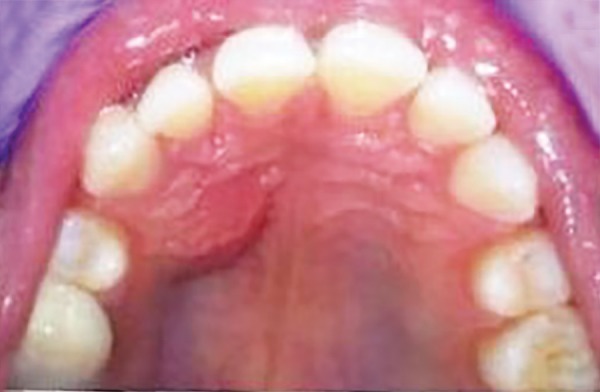
Preoperative photograph

**Fig. 2:**
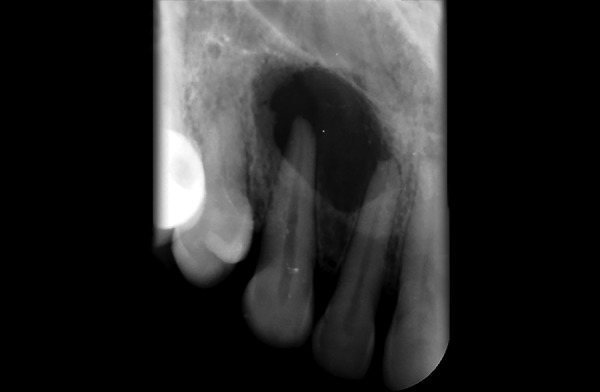
Intraoral periapical w.r.t. 12,13 showing periapical radiolucency

**Fig. 3:**
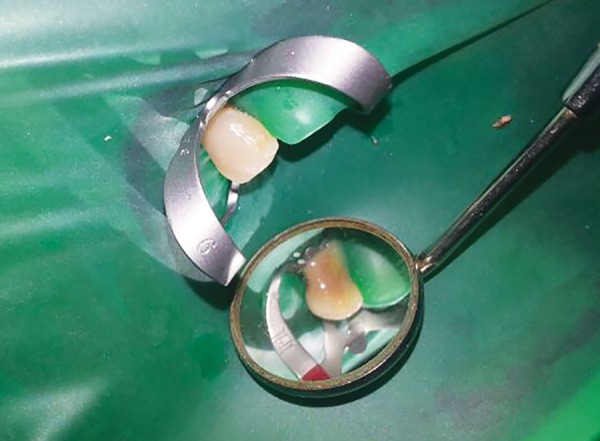
Emergency access opening with pus discharge

**Fig. 4:**
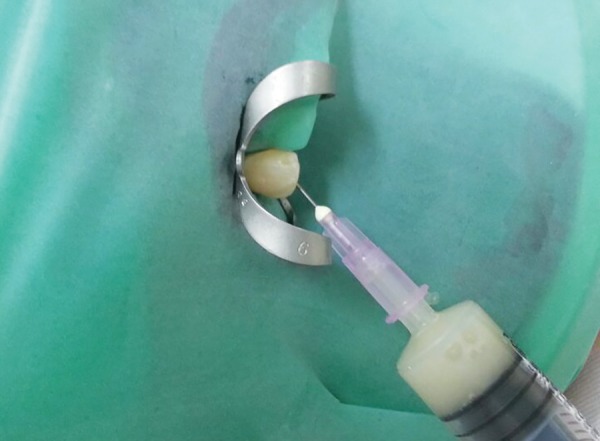
Aspiration of pus

**Fig. 5:**
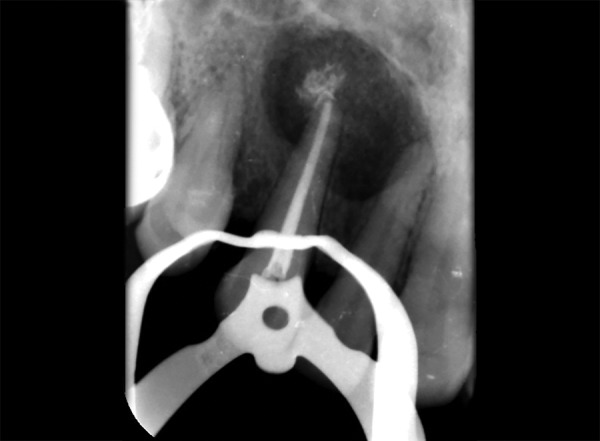
Metapex intracanal dressing

**Fig. 6:**
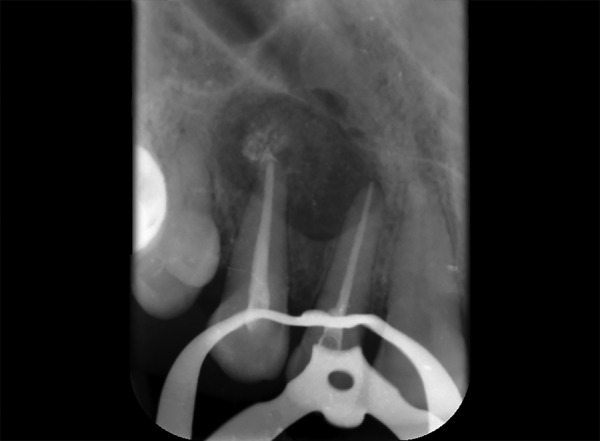
Obturation w.r.t. 12,13

**Fig. 7:**
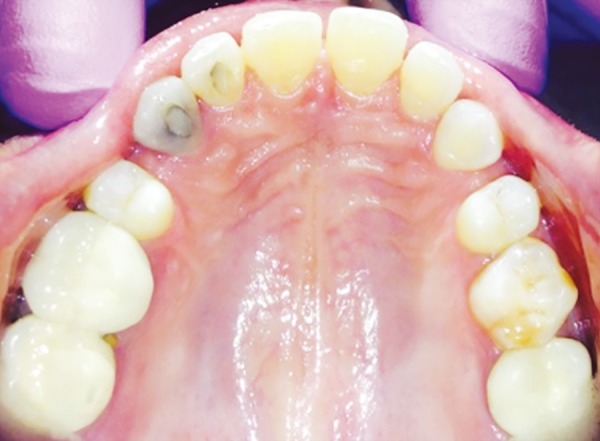
Postoperative photograph

**Fig. 8:**
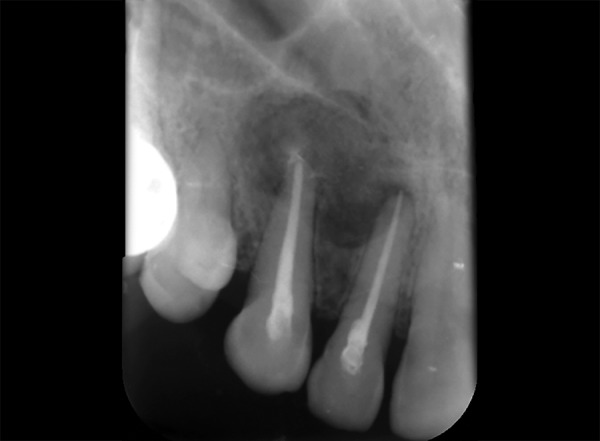
Eighteen months postoperative radiograph

A 20 years old female patient reported to the dental clinic with the chief complaint of a large palatal swelling. The patient gave a history of trauma to her anterior teeth when she was 17 years old. At that time, she did not seek any treatment for the same. On examination, a palatal swelling was seen behind the canine (Fig. 1). Lymph nodes were nonpalpable. Vitality testing was done w.r.t. 13, 12 in which 13 was nonvital and 12 gave delayed response. Hot and cold tests were done in which again no response was elicited w.r.t. 13 and delayed response w.r.t. 12. The swelling was 2 × 2 cm in size. The tooth (13) was slightly discolored. On radiographic examination, a large well circumscribed radiolucency was seen involving the roots of canine and lateral incisor (Fig. 2). Slight displacement of roots was also seen. Patient was given antibiotics at the beginning of the treatment and then the abscess was punctured after 1 week. When the root canal was opened i.r.t. 13, suppurative fluid mixed with blood (Fig. 3) followed which was aspirated and sent for culture and examination (Fig. 4). Positive aspiration was done in subsequent visits and intermediate open dressings were given for 2 weeks i.r.t. 13. Once the canal was completely dried and no pus discharge was seen, Ca(OH)_2_ (metapex) dressing was given (Fig. 5) and changed regularly for the next 8 weeks. Vigorous warm saline irrigation was done initially and later the canal was irrigated with 2.5% hypochlorite. Simultaneously, root canal treatment i.r.t. 12 was also started and no pus discharge was seen. Obturation for both the teeth was done after 6 months (Fig. 6). The patient was followed for next 18 months and good healing was observed clinically and radiographically (Figs 7 and 8).

### Case 2

**Fig. 9:**
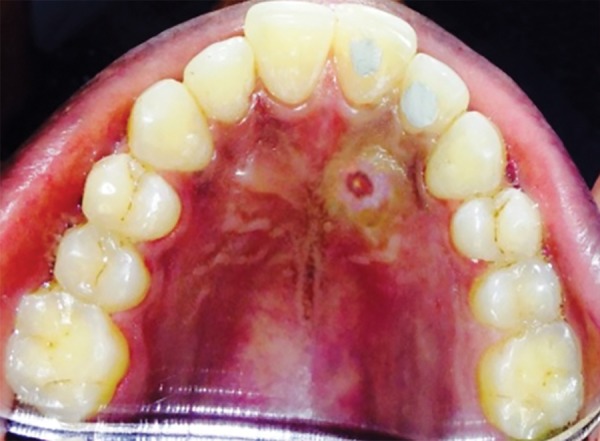
Preoperative photograph

**Fig. 10:**
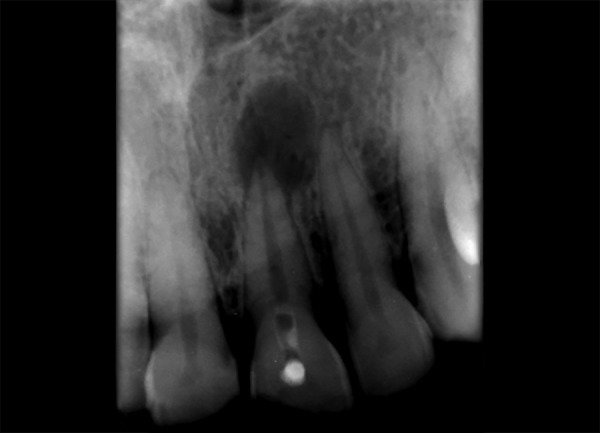
Preoperative IOPA w.r.t. 21, 22

**Fig. 11:**
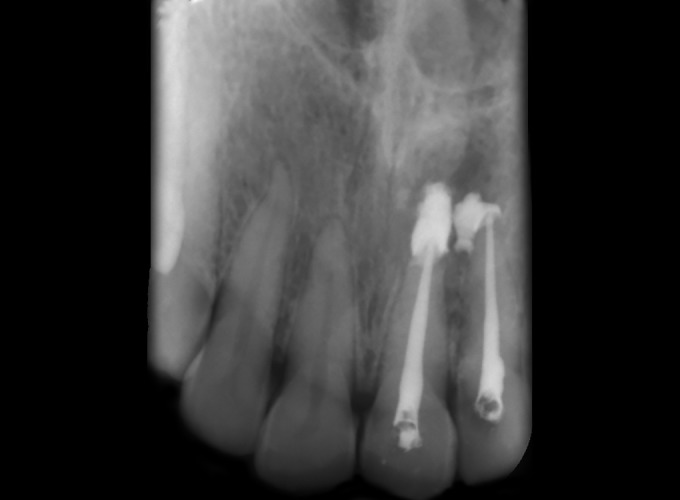
Metapex intracanal medicament

**Fig. 12:**
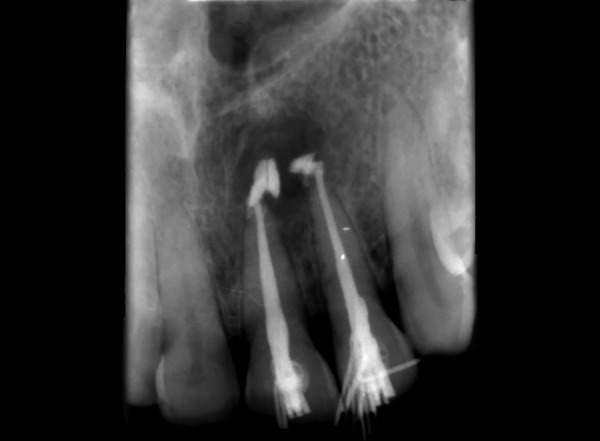
Obturation w.r.t. 21, 22

**Fig. 13:**
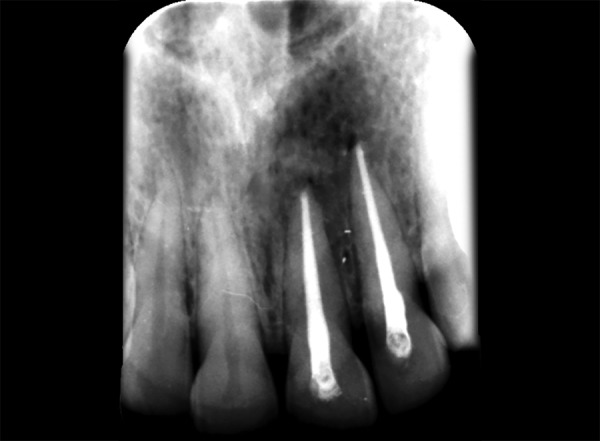
Eighteen months postoperative radiograph

**Fig. 14:**
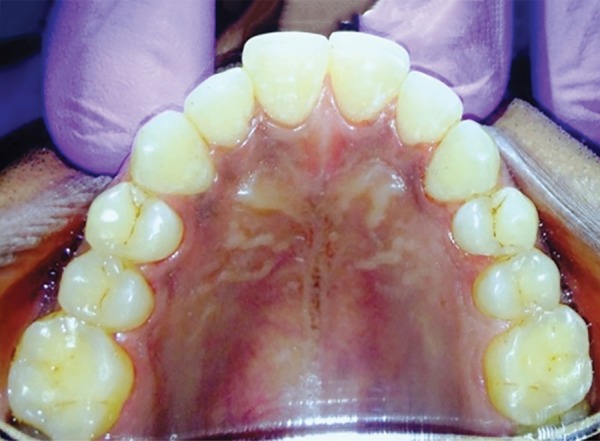
Eighteen months postoperative photograph

An 18 years old female patient reported to the dental clinic with the chief complaint of pain and in the upper right tooth region and a palatal swelling. Patient gave a medical history of asthma. Patient gave a dental history of incomplete root canal 10 years back i.r.t. 21. At that time patient did not seek any further treatment. On examination severe tenderness was seen w.r.t. 21, 22. Palatal swelling was seen in the rugae area with a profound stoma present (Fig. 9). Discoloration was seen w.r.t. 21. Vitality tests were done and 21 was found to be nonvital and delayed response was seen in 22. Hot and cold test were also done in which 21 was negative and 22 gave a delayed response. Lymph nodes were non palpable. Radiograph was done in which a large periapical radio-lucency was seen w.r.t. 21, 22 (Fig. 10). Emergency access opening was done without the rubber dam as the patient was asthmatic. A thick purulent discharge followed. Similar irrigation protocol and positive aspiration was followed for this patient as well. Calcium hydroxide was given as intracanal medicament for next 8 weeks (Fig. 11). The dressing was changed regularly. Metapex dressing was removed and then the teeth were obturated after 6 months (Fig. 12). Further the patient was followed for 18 months postoperatively and radiographically good healing was seen (Fig. 13). On clinical examination after 18 months, no palatal swelling was seen and no discomfort was reported by the patient (Fig. 14).

## DISCUSSION

The treatment options for large periapical lesions may range from conventional nonsurgical root canal therapy with calcium hydroxide intracanal medication to various surgical interventions.^7^ The precise mechanism involved in the formation of periapical lesions is not fully understood. Nevertheless it is generally agreed that if the pulp becomes necrotic, its environment becomes suitable to allow microorganisms to multiply and release various toxins into the periapical tissue initiating an inflammatory reaction and leading to the formation of periapical lesion.^8^ Toller (1972) proposed that the growth of the cyst may be attributable to the increased hydrostatic pressure of the confined fluid, which causes additional osteoclastic activity.^9^

Calişkan stated that in approximately 70% of cases with periapical lesion, the healing was apparent within 2 years of treatment.^10^ In the present cases, the teeth were obturated after 6 months and were further followed up for next 18 months. After which complete healing was seen.

Metapex, a silicone oil-based calcium hydroxide paste containing 38% iodoform is very popular intra-canal dressing. Metapex contains silicone oil as its vehicle and has a pH below that which is effective to kill *Enterococcus faecalis.* The superior antimicrobial effects of Metapex may be due to the combination with iodoform and to the viscous and oily vehicle, which may prolong the action of the medicament. Accordingly, Gomes et al showed that oily vehicles increase the antimicrobial effects of calcium hydroxide against *E. faecalis* and other bacteria.^11^ Calcium hydroxide is an intracanal medicament that is commonly used because of its ability to predictably disinfect and neutralizes remaining microorganisms in the root canals. The mechanisms of Ca(OH)_2_ are not fully understood. Additionally, its biological properties are achieved by the dissociation in Ca^2+^ and OH^‒^ ions.^12^ The antimicrobial effects of Ca(OH)_2_ relate directly to its high pH 12.5, it has a destructive effect on cell membranes and protein structures. Because it plays a major role as an inter appointments dressing in the disinfection of the root canal system, a Ca(OH)_2_ based paste was used as an antibacterial dressing in this case.^13^ An important aspect of the irrigation procedure is to ensure that there is an escape wound for the saline to drain. Gentle irrigation would cleanse the cavity and saline will not be forced into the surrounding tissues.^14^ Additionally, increasing the temperature of saline has shown to be more effective in breaking the loculi and releasing the pressure. Sodium hypochlorite was not used for initial irrigations as it results in coagulation of the pus.^14^

The aspiration of fluid was accompanied by the application of digital pressure so that the swelling decreases in size. Positive aspiration resulted in negative pressure and which helped in releasing the pressure from the bony cavity. It is important to apply digital pressure even whilst the canal is being dried and temporized to avoid entrapment of air in the bony cavity, which can result in an increased intrabony pressure once the tooth is temporized.^15^

The advantages of nonsurgically managing patients with large periapical radiolucencies is that the psychological trauma is less and is more comforting to the patient. The periapical lesions in both patients were large and after nonsurgical endodontic therapy the lesions completely resolved. The periapical tissues have a rich blood supply, lymphatic drainage, and abundant undifferentiated cells. All these structures are involved in the process of inflammation and repair. Therefore, because the periapical tissues have the potential to heal, treatment of periapical lesions should be directed toward only removal of the causative factors.

## CONCLUSION

A nonsurgical approach should always be adopted before resorting to surgery. The decompression and aspiration-irrigation techniques can be used when there is drainage of cystic fluid from the canals. These techniques act by decreasing the hydrostatic pressure within the periapical lesion. Regular change of intracanal dressings of metapex has proved to be very beneficial for reducing the size of periapical lesion.
